# Chinese validation of “subjective motoric cognitive risk syndrome” screening tool in patients with coronary artery disease using Rasch analysis

**DOI:** 10.3389/fragi.2025.1505847

**Published:** 2025-05-15

**Authors:** Yiyi Chai, Yanrong Gu, Xiaomin Wu, Yini Wang, Ping Lin, Qingfang Ye, Ling Li

**Affiliations:** ^1^ Department of Nursing, Harbin Medical University, Harbin, China; ^2^ Department of Cardiology, The Second Affiliated Hospital of Harbin Medical University, Harbin, China; ^3^ Department of Basic Nursing, School of Nursing, Harbin Medical University, Daqing, China

**Keywords:** motoric cognitive risk syndrome, coronary artery disease, Chinese validation, Rasch analysis, subjective cognitive complaint

## Abstract

**Objective:**

Subjective motoric cognitive risk syndrome (MCR-S) is a well-established screening tool that has been validated for objective motoric cognitive risk syndrome (MCR-O) and predicted risk of incident dementia. MCR is associated with cardiovascular factors and coronary artery disease (CAD). MCR-S is crucial for remote cognitive screening but has only been validated in community settings so far. Our study aimed to validate a Chinese version of the MCR-S in CAD patients.

**Method:**

The Chinese version of the MCR-S was obtained through a standardized forward-backward translation and cultural adaptation. 338 CAD patients were recruited. Traditional analysis based on classical test theory and Rasch analysis based on latent trait theory were performed on the MCR-S for validation. Receiver operating characteristic analysis was applied to determine the discriminative ability of MCR-S for the MCR-O in CAD patients.

**Results:**

The MCR-S met the unidimensionality, lack of local dependency or disordered thresholds, and good fit value for each item of the Rasch model, the item-person map shows that the item’s estimate of capacity is appropriate. MCR-S has good content validity, criterion-related validity, and test-retest reliability. An optimal cut-score of 4.6 on the MCR-S score was determined to have good sensitivity (79.2%) and specificity (71.3%) for MCR-O in CAD patients.

**Conclusion:**

The Chinese version of MCR-S meets the requirements of the Rasch model and has good validity in CAD patients. The validated MCR-S cutoff can support long-term monitoring and early intervention for CAD patients at risk of MCR-O.

## 1 Introduction

MCR is a predementia syndrome marked by cognitive complaints and slow gait, serving as an early predictor of Alzheimer’s and vascular dementia. (Verghese et al., 2013). Existing evidence demonstrates that cognitive complaints and slow gait are associated with chronic diseases such as atherosclerosis and cardiovascular burden (Jorm et al., 2004; Welmer et al., 2013; Heiland et al., 2017); coronary artery disease (CAD) is significantly associated with cognitive impairment and dementia (Deckers et al., 2017; Wolters et al., 2018). The correlation between MCR and CAD is flanked by evidence of MCR and cardiovascular disease and its risk factors (Zhang et al., 2023; Iqbal et al., 2022). And there is also direct evidence to support a correlation between MCR and CAD (Chhetri et al., 2020a). CAD is the most prevalent chronic disease in the world, and early recognition of the onset of MCR in patients with CAD, as well as dynamic detection of MCR changes, are critical for controlling the progression of the disease and improving the quality of survival in later life. However, MCR limits remote observation and management efforts because it requires field measurements of objective gait decline.

Individuals may perceive changes in their cognitive abilities or walking patterns before developing objective cognitive or motoric impairments (Ferrucci et al., 2016; Studart and Nitrini, 2016). The Subjective Motoric cognitive risk syndrome (MCR-S) is a scale tool developed based on objective MCR (MCR-O) research, which contains subjective motoric complaint (SMC) and subjective cognitive complaint (SCC), replacing the need for an objective measure of gait speed with SMC. The MCR-S has demonstrated both concurrent validity for the MCR-O and predictive validity for dementia (Ayers et al., 2022). In addition to effectiveness, the MCR-S saves time and labor costs and offers great convenience. The MCR-S scale consists of only five items and can be administered in less than 5 min and can be quickly and easily administered remotely (longitudinal telephone follow-up) by a non-clinician (Ayers et al., 2022).

China is a populous country with a heavy healthcare burden, and the prevalence of cardiovascular disease and dementia is on the rise due to aging, requiring a more convenient method for early identification of cognitive decline, which provides a practical necessity for the Chinese localization of the MCR-S. - Previous studies have validated the MCR-S in community-residing American adults aged ≥65 years (Ayers et al., 2022) and in German adults aged 50–82 years through an online survey (Theobald et al., 2025), lack of validation in LMICs contexts and middle-aged and elderly CAD population. Although population databases in Taiwan have been investigated, the MCR-S remains unvalidated in local populations (Chuang et al., 2024). Therefore, the generalizability of the MCR-S in middle-aged and elderly patients with CAD requires further validation in regionally representative cohorts.

The MCR-S is a questionnaire containing five dichotomous items. Traditional validity and reliability analysis based on classical test theory (CTT), and some analytical techniques such as factor analyses were developed for continuous data, which is unsuitable for dichotomous data (Clark and Bowles, 2018). The Rasch model is an analytical model used to evaluate the measurement properties of rating scales using probability estimates based on latent trait theory (LTT), which is complementary to CTT (Rasch, 1980). The Rasch model is the simplest one-parameter model of the item response theory (IRT) measurement model for dichotomous. Rasch analysis overcomes the limitations of CTT and can capture the respondent’s ability and the item’s difficulty with the same metric. The model estimates item location and person location separately on a common interval level logit (log-odd units) scale. If data fit the model, then linear measurement and invariant comparisons are possible. In recent years, the Rasch analysis has been increasingly used to assess measures of health outcome (Sun et al., 2021; Alenius et al., 2024).

Our study aimed to translate the MCR-S into Chinese and fully validate the Chinese version in patients with CAD by Rasch analysis based on LTT and traditional analysis based on CTT. Our survey may contribute to provide evidence for further revision and refinement of the MCR-S as well as future studies of the relevance of the MCR in the field of CAD and other chronic diseases.

## 2 Methods

### 2.1 Cross-cultural adaptation

Our study followed published recommendations and guidelines for translation and cross-cultural adaptation (Brislin, 1970; Beaton et al., 2000).

The translation process followed a structured approach (flowchart see Supplementary Table S1).

Step 1: Direct translation by two independent translators
Step 2: Synthesis of both translations into one version
Step 3: Back-translation by two native English speakers
Step 4: Review by a research committee
Step 5: Expert consultation for cultural adaptation
Step 6: Pretesting with CAD patients

The English version and Chinese version of MCR-S are shown in Supplementary Table S2.

In our study, we found that the conversion of 1/4 mile to 400 m, according to the direct translation, did not match the context and our selected patient’s abilities. We found that self-reported difficulty walking 1 km was used to measure mobility disability. This distance is closest to one-quarter of a mile, which is a common mobility question in the United States (Capistrant et al., 2014). Therefore, we translate the first item in SMC as *How far can you walk in an hour? <1 km.*

### 2.2 Study population

According to prior Rasch research, a sample size of approximately 250 participants yielding 99% confidence with a stable item calibration within ±0.5 logits provides a stable model (Linacre, 1994; Guilleux et al., 2014). Between September and December 2023, 350 patients with CAD participated in the survey, 12 patients who did not meet the criteria were excluded, and finally, 338 patients were included in this study (see Supplementary Figure S1). A sample size of 338 was determined based on a power analysis with an effect size of 0.89, α = 0.05, and power = 0.80, ensuring sufficient statistical precision for ROC curve validation, the effect size was selected from the AUC values of the original study of MCR-S (Ayers et al., 2022). The inclusion criteria were as follows: diagnosed with CAD by the attending physician, older than 45 years of age (according to the World Health Organization and the China Health and Retirement Longitudinal Study standards of middle age and older). The exclusion criteria were as follows: heart failure, myocardial infarction, visual disorders, hearing disorders, mobility disability (difficulty when dressing or eating, walking across a room, bathing, or showering, getting in or out of bed, and using the toilet) (Katz et al., 1963), self-reported diagnosis of Alzheimer’s disease, dementia, Parkinson’s disease, and being younger than 45 years old.

We adhered to the principles of the Declaration of Helsinki and the study protocol was approved by the Ethics Committee of the Second Affiliated Hospital of Harbin Medical University, the ethics number is YJSKY2023-349. All participants gave written informed consent.

### 2.3 Motoric cognitive risk syndrome diagnosis

The motoric cognitive risk was defined using established criteria such as the presence of SCC and slow gait velocity in older individuals without dementia or mobility disability (Verghese et al., 2013). We excluded participants who were diagnosed with dementia by the MMSE: (i) illiteracy and MMSE score <17; (ii) primary school education and MMSE score <20; and (iii) middle school and higher education and MMSE score <24. Cognitive complaints were assessed by the presence of the following: a “yes” response to the memory item on the Geriatric Depression Scale (GDS): “*Do you feel you have more problems with memory than most?*“ (Yesavage et al., 1982). Gait speed was measured using the 4-m walking velocity as an objective measurement. The participants were instructed to stand behind the start line with their toes touching the start line and to walk the whole distance at their usual pace until past the finish line. The average time of two trials was used to compute gait speed (Zhang et al., 2020). Slow gait was defined as walking speed one standard deviation (SD) or more below age- and sex-specific means (Verghese et al., 2013; 2014). The cut-off values for defining slow gait for different age groups (45–54, 55–64, 65–74, and ≥75 years old) in our cohort were 78, 72, 67 and 55 cm/s in males and 72, 65, 59 and 40 cm/s in females, respectively.

### 2.4 Statistical analysis

#### 2.4.1 Rasch analysis

Rasch analysis were performed with Winsteps software (version 3.72.3; https://winsteps.com/index. htm) and R (version 4.4.0). An outcome measure should fulfill the Rasch model expectations such as unidimensionality, lack of local dependency or disordered thresholds.

Unidimensionality: Rasch residual-based principal component analysis (PCA) was used to assess unidimensionality. An eigenvalue of the first contrast of residuals smaller than 2.0 and more than 50% variance explained by the measures are considered to support unidimensionality (Linacre, 1994).

Local dependency: When participant item responses depend not just on their trait level, but on their responses to other test items, this can indicate local dependency. Inter-item residual correlations > 0.3 above the average residual correlation indicate local dependency.

Item threshold: The point between adjacent response categories where both responses are equally probable is the item threshold. If people struggle to distinguish between response options, the test’s accuracy is reduced. Item fit: Rasch model for each item on the questionnaire is assessed by mean square (MNSQ) error such as the information-weighted fit (INFIT) and the outlier-sensitive fit (OUTFIT). An acceptable range of values for the INFIT MNSQ and the OUTFIT MNSQ is 0.5–1.5 (Linacre, 1994).

Separation index and reliability: Separation refers to the number of statistically different performance strata that the test can identify in the sample. A person separation index (PSI) above 2.0 implies that the instrument is sensitive enough to distinguish between high and low performers and an item separation index (ISI) above 3.0 implies that the person sample is large enough to confirm the item difficulty hierarchy (construct validity) of the instrument. Person reliability indicates the replicability of person ordering expected if the sample were given another set of items measuring the same construct, whereas item reliability specifies the replicability of item placements along the scale if these same items were given to another same-sized sample with similar knowledge levels. Values above 0.8 for persons and items are generally considered satisfactory (Bond and Fox, 2015).

Item-person map: The Rasch Item-Person Map is to determine whether the distribution of items along the logit scale of difficulty approximately mirrored the distribution of respondent ability. Larger logit values in the scale indicate higher trait levels or greater item difficulty (McAlinden et al., 2017).

IIC and TIC: The Item Information Curve (IIC) and Test Information Curve (TIC) reflect the relationship between the information contribution of different items/full scale in assessing a subject’s latent trait level. The peak of the curve represents the maximum amount of information that can be provided when the subject’s level of latent traits best matches the difficulty of the item.

#### 2.4.2 Reliability and validity based on CTT

Data were analyzed with Statistical Product and Service Solutions (SPSS) (version 25).

Reliability: Correlation coefficients was performed to assess test–retest reliability for a second administration of the MCR-S, and 50 participants (15%) completed a second copy of the questionnaire within 2–3 weeks for the test-retest assessment (Park et al., 2018).

Validity: Validity was tested using content validity and criterion validity. Content validity is whether the domain of content for the construct is adequately represented by the items. The item content validity index (I-CVI) is calculated by counting the number of experts who rated the item as three or four and dividing that number by the total number of experts (ten experts in the field). The average content validity index (Ave-CVI) is summing the I-CVIs and dividing them by the number of items (Almanasreh et al., 2019). The I-CVI was considered acceptable for values > 0.78 and the Ave-CVI for values > 0.80 (Polit et al., 2007). The criterion-related validity was assessed by calculating the Pearson correlation coefficient with the Subjective Cognitive Decline-Questionnaire 9 (SCD-Q9). Receiver operating characteristic (ROC) analysis was applied to evaluate the criterion validity of the MCR-S risk score for MCR-O. The area under the curve (AUC) was calculated to quantify the screening ability of the MCR-S. In addition, the cut-off point was empirically calculated with Youden’s index (J) to define MCR-S.

## 3 Result

### 3.1 Demographics

At baseline, 24 (7.1%) participants were diagnosed with MCR-O. The demographic and clinical characteristics of all participants are summarized (see Table 1).

**TABLE 1:** Demographic characteristics (N = 338).

Characteristics	Value
Age, years, mean ± SD	63.7 ± 8.0
Sex, n (%)	
Male	189 (55.9)
Female	149 (44.1)
Education, n (%)	
Elementary school and below	118 (34.9)
Middle school	181 (53.6)
College and junior college	39 (11.5)
MCR-S total score, mean (SD)	3.4 (1.7)
Item1, (yes) n (%)	130 (38.4)
Item2, (yes) n (%)	156 (46.1)
Item3, (yes) n (%)	282 (83.4)
Item4, (yes) n (%)	40 (11.8)
Item5, (yes) n (%)	120 (35.5)
Four-meter usual gait speed, mean (SD)	
Male	0.86 (0.18)
Female	0.79 (0.19)

SD, standard deviation.

### 3.2 Rasch analysis

The analysis of unidimensional: The first contrast eigenvalues remained below 2.0, confirming unidimensionality. (see Supplementary Table S1). The analysis of local dependency: All inter-item residual correlations < 0.3, which indicates that the scale satisfies the Rasch model’s lack of item dependence (see Supplementary Table S2).

The analysis of item threshold: The observed average and category measure values both show monotonically increasing, which indicates that the scale satisfies the Rasch model’s lack of disordered thresholds. individual category probability curves. Thresholds could review the individual category probability curves (see Supplementary Figure S2).

The analysis of item fit: The range of scale item difficulty was −3.37–2.74 logit scores, and the standard error of the difficulty estimate for each item was 0.14–0.22. The item infit statistics ranging from 0.88 to 1.12 and the outfit statistics ranging from 0.80 to 1.33. The infit/outfit statistics of the remaining entries were within 0.5–1.5, which fit the model. The PT-Measure Corr is the correlation between the patient’s score on the question and his or her total score on the measure, reflecting the discriminatory effect of the item. The PT-Measure Corr ranging from 0.43 to 0.66, all above 0.4, indicating good discriminant validity of the questions (see Table 2).

**TABLE 2:** Rasch model fit statistics item locations, fit residuals.

Item	Location	S.E	INFIT		OUTFIT		PT-measure Corr
			MNSQ	ZSTD	MNSQ	ZSTD	
TIQ1	0.31	0.14	1.04	0.6	0.98	−0.1	0.58
TIQ2	−0.20	0.14	0.88	−1.9	0.80	−2.1	0.66
TIQ3	−3.37	0.21	0.99	0.0	0.96	0.1	0.59
TIQ4	2.74	0.22	1.12	0.9	1.33	0.9	0.43
TIQ5	0.52	0.14	0.99	−0.1	1.21	1.6	0.57

*OUTFIT, outlier-sensitive fit; INFIT, information-weighted fit; MNSQ, mean square; ZSTD, standardized fit statistics. Infit means inlier-sensitive or information-weighted fit. Outfit means outlier-sensitive fit. Mean-square fit statistics show the size of the randomness*. *Standardized fit statistics are t-tests of the hypothesis*. *In a Rasch context they indicate how accurately or predictably data fit the model*.

The analysis of separation index and reliability: The items had a large separation index (15.61) while the persons had a small separation index (1.03); great item reliability (1.00), and insufficient person reliability (0.52) (see Supplementary Table S3). This indicates that the items have good differentiation and match the ability level of the subjects well. However, the distribution of subjects’ abilities is not broad enough and the differentiation needs to be improved.

**FIGURE 1:**
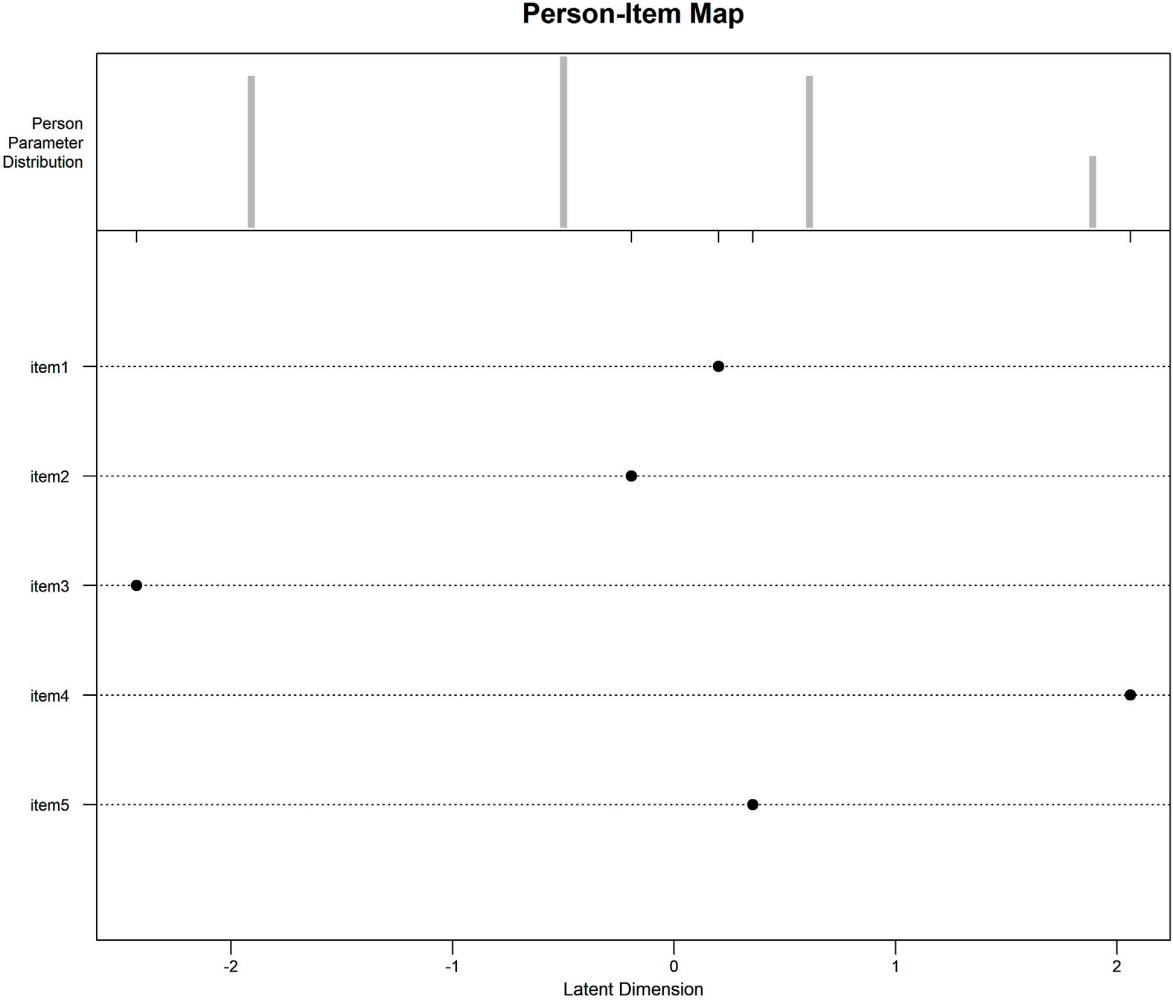
Item-Person Map: The Item-Person Map compares subjects’ trait levels and difficulty of items on the same Logit scale, with the Logit scale at the bottom and the measurements progressively higher from left to right. The top half is the individual trait levels, and the height of the bar graph indicates the number of subjects in this position; the more dispersed the distribution of subjects, the higher the scale differentiation. The lower half of the bar indicates the difficulty of the questions. As can be seen from Figure 1, individual ability is approximately normally distributed, the number of subjects with medium ability is the largest, but the distribution is relatively concentrated, indicating that the smaller the differentiation of the scale; item four is the most difficult, and item three is the easiest, and the distribution of the difficulty of the items is broader, covering subjects of all trait levels.

The analysis of the item-person map: The overall distribution of the items shows that the five items have a wide range of difficulty, which can be evenly dispersed and cover patients of all abilities, with the hardest topic being Q4 and the easiest topic being Q3. The height of the bars in the graph indicates the number of subjects located in this position, and the distribution of subjects is close to normal, i.e., there are more subjects of intermediate ability, but the distribution is more dispersed, indicating that the sample is not sufficiently differentiated. (see Figure 1).

**FIGURE 2:**
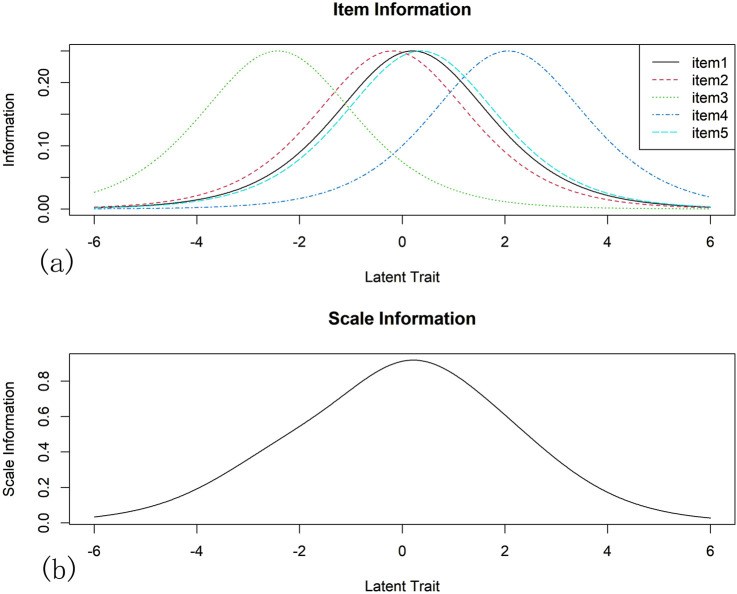
Item Information Curve (IIC) and Test Information Curve (TIC): The IIC and TIC reflect the relationship between the information contribution of different items/full scale in assessing a subject’s latent trait level. The difficulty of the item can be seen as the horizontal coordinate, representing the trait level of the subject, and the vertical coordinate representing the amount of information. The peak of the curve represents the maximum amount of information that can be provided when the subject’s level of latent traits best matches the difficulty of the item. IIC is shown in **(a)**. The maximum information provided by item 1, item 2, and item five were near its average calibration. Item three provides the most information for low ability subjects and item four provides the most information for high ability subjects. TIC is shown in **(b)**. The greatest amount of information was found when respondents reported trait levels close to the item-calibrated mean (logit = 0), with the overall scale providing the greatest amount of information for moderately competent subjects.

The analysis of IIC and TIC: IIC is shown in Figure 2a. The maximum information provided by item 1, item 2, and item five were near its average calibration. Item three provides the most information for low ability subjects and item four provides the most information for high ability subjects. TIC is shown in Figure 2b. The greatest amount of information was found when respondents reported trait levels close to the item-calibrated mean (logit = 0), with the overall scale providing the greatest amount of information for moderately competent subjects.

### 3.3 Validity and reliability

**FIGURE 3:**
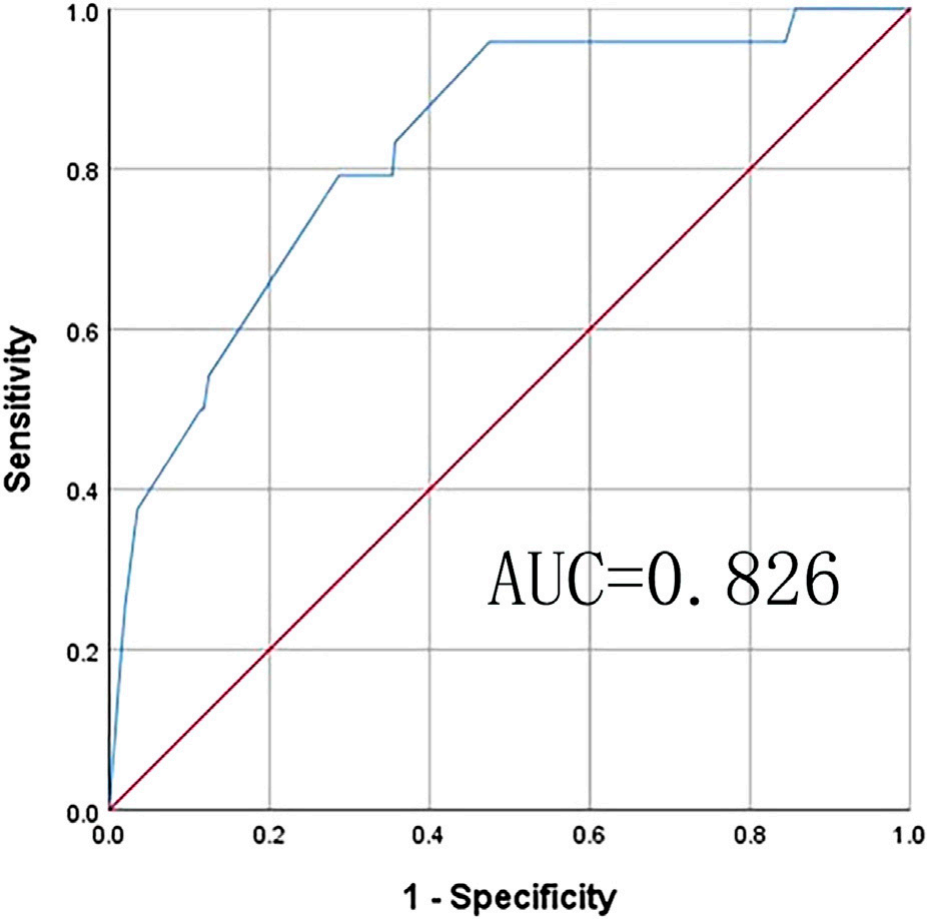
Receiver operating characteristic (ROC) analysis: ROCfor MCR-S sensitivity and specificity for objective MCR. Area under curve (AUC) is the area under the ROC curve that is enclosed by the axes, and the value of AUC ranges between 0.5 and 1. AUC values of 0.5–0.7, 0.7–0.9, and >0.9 indicate low, moderate, and high accuracy, respectively. Youden’s index (J) gives the difference between the true positive rate and the false positive rate (sensitivity + specificity–1). This index ranges from 0 to 1, with values closer to 0 indicating limited effectiveness, and those near one suggesting that the overall effectiveness of a cut-off point is relatively large.

The Chinese MCR-S showed excellent test–retest reliability, with an intraclass correlation coefficient of 0.92. The item-CVI was 1.00 for Items 1, 4, and 5, and 0.90 for Items 2 and 3. The average scale-CVI was 0.96, suggesting excellent content validity. The final Chinese version of the MCR-S was moderately correlated with the SCD-9 (0.70). The ROC curve for the MCR-S score in discriminating MCR-O at baseline. The AUC was 0.826 (95% CI 0.743–0.909) with an optimal cut-score of >4.6 determined by the Youden index, this MCR-S cut-score yielded a sensitivity of 79.2% and specificity of 71.3%for MCR-O (see Figure 3).

## 4 Discussion

We successfully translated the MCR-S into Mandarin Chinese, adapted it to Chinese culture, and validated this adaptation. Overall, the scale has good validity and external reliability.

### 4.1 Validity and reliability

The Rasch model, grounded in LTTs, posits that the probability of an individual’s response to a rating scale category depends on the discrepancy between their ability level and the item’s difficulty parameter (Hobart et al., 2007). Unlike other item response theory (IRT) models (e.g., 2PL or 3PL), which incorporate multiple sample-dependent parameters, the Rasch model employs a single parameter to evaluate how closely observed responses align with theoretically predicted patterns (Andrich, 2004). Specifically, Rasch Analysis allows measurement on an interval scale, ensuring unidimensionality and detecting item misfit, which is especially useful for dichotomous response data like MCR-S. Item Characteristic Curve (ICC) of this scale are presented in Supplementary Material (see Supplementary Figure S3). The scale is consistent with the model’s three prerequisite assumptions, which are unidimensionality, lack of local dependency, and lack of disordered thresholds. PCA on the standardized residuals revealed that the MCR-S was a unidimensional scale, with the scale meant to measure one, rather than several, potential qualities, namely, the ability to exercise the cognitive risk syndrome. Rasch analysis confirmed a good fit for each item, indicating the scale measures the intended trait without external influence. The Item-Person map illustrates that the item’s capacity estimate is accurate. As with classical test theory, reliability values greater than 0.8 are desired, although this rule of thumb must be balanced against the sample’s heterogeneity and the objective of the test. Person reliability will be higher in heterogeneous samples than in samples where everyone has a similar value of the latent characteristic. It will also be higher with longer tests than with shorter tests since the latent characteristic values will be estimated more precisely. Lower person reliability may be due to the study population’s lack of heterogeneity (all hospitalized patients with CAD) and the limited number of items.

The item-CVI indicates outstanding content validity. Our findings also support the discriminative validity of MCR-S in screening for MCR-O in a CAD-based cohort of persons over the age of 45. A cutoff score of 4.6 demonstrated strong sensitivity and specificity for detecting MCR-O in CAD patients. Of the 338 eligible subjects, 109 (32.2%) exceeded the MCR-S cutoff of >4.6 at baseline. The exterior reliability was tested using test-retest reliability, and the findings were satisfactory. The moderate association with SCD-Q9 indicates moderate external validity, which could be attributed to the fact that SCD-Q9 only investigated characteristics of SCC. The traditional reliability and validity are obtained from CTT. The CTT hypothesis states that a person’s rating scale score (O) is the total of the unobservable measurement to be estimated (T) plus the accompanying measurement error (E). Credibility and validity are a continuation of two distinct research orientations: quantitative, organized approaches and qualitative, ideographic methods. The first is more credible, whilst the second is more valid.

### 4.2 Discussion of items

MCR syndrome combines these two early harbingers of dementia, and the likelihood of cognitive decline or dementia is greater for MCR than for either slow gait speed or subjective memory complaint alone (Semba et al., 2020). The MCR-S contains SCC (3 items) and SMC (2 items), each of the five items is discussed below.

Attention is a characteristic and property of multiple perceptual and cognitive control mechanisms (Chun et al., 2011). Attention and concentration is a multifaceted construct and is generally divided into two global subdomains: selective attention and sustained attention (or vigilance). Concentration would generally fall under the rubric of sustained attention (Harvey, 2019). Sustained attention is sometimes considered as a core executive function. Item one examines the concept of sustained attention (Staub et al., 2013). We discovered in our actual survey that concentration is an abstract term for some people, requiring adequate explanations from the investigator. When asked about items, many patients volunteered to describe signs of a change in their memory capacity or ability with daily problems, such as forgetting the names of acquaintances, forgetting where they put things, and so on, or to elaborate on whether they had a recent or distant memory loss (AD patients often start to show a progressive decay of working memory, which is a special kind of short-term memory (Jahn, 2013)). 83% of the population replied yes to question 3, with 74% even among middle-aged persons aged 45–60 years, indicating the importance and urgency of identifying patients with CAD who are at risk of cognitive impairment. However, they are reserved in answering item two of whether they have more problems with your memory than most, which is a psychological variable that deserves our attention in the elderly. In addition, the most of the entry should be clear whether it is a group of the same age or one that includes young people, because conceptual ambiguity can lead to greater variability in responses. The diagnostic criteria for SCC in the MCR-O changed later, from the original *“yes” response to “Do you feel that you have more problems with your memory than most?“(memory item on the GDS)* change to: *a score of ≥ 1 on the AD8 dementia screener or a “yes” response to the memory item on the GDS* (Ayers et al., 2022). Item2 of the MCR-S is the memory item on GDS and item3 of the MCR-S is an item from AD8, and when we refer to both items to determine the SCC of MCR-O, the incidence of MCR-O rises to 11.6%, but the validation effect of the MCR-S on MCR-O decreases (AUC = 0.729), and the specificity of the cutoff value to be small. Because more than half of the studies used initial approach to diagnose SCC (Nester et al., 2024), we used only item two in this article to determine MCR-O. The criteria for judging SCC in MCR-O are controversial, should be standardized in future studies (Nester et al., 2024).

SMC is a new concept, which has been proven a harbinger of mobility disability and can help improve clinical risk assessments and identify high-risk individuals for interventions to prevent onset of slow gait (Verghese and Ayers, 2020). In the pre-experiment, we found that almost all of the patients were capable of walking 400 m in 1 h if 1/4 mile was directly translated into 400 m, which is extremely inconsistent with the probability of answering yes to item four in the original survey (11.1%). Through expert consultation and literature review, we found that 1/4 mile is a common mobility question in the USA, self-reported difficulty walking 1 km is the question used by WHO Disability Assessment Scale and China Health and Retirement Longitudinal Study. In low- and middle income countries (obtain China), self-reported difficulty walking 1km was validated to have a generalized correspondence with objective step mobility (Capistrant et al., 2014). In addition, we did the correlation analysis and logistic regression between the changed item four and the customized slow gait, as well as the logistic regression between item four and MCR-O, respectively, and the results showed that P < 0.01. It can be justified that the improvements we have made are reasonable. It would be more scientific to develop items that match the Chinese population. Item four and item five are about the examination of athletic endurance. The answer to the item of whether climbing stairs is difficult is also relatively ambiguous, as patients’ self-perceived difficulty will vary relative to different numbers of steps/floors, which also appeared in the original authors’ previous study (Verghese et al., 2008; Oh-Park et al., 2011).

### 4.3 Application

Emerging evidence underscores a robust association between MCR and CAD. Recent epidemiological investigations reveal that cardiometabolic multimorbidity significantly elevates MCR risk (Zhang et al., 2023), with a dose-dependent relationship observed between the cumulative burden of cardiometabolic disorders and incident MCR (She et al., 2024). Mechanistic studies further suggest that cardiometabolic-vascular pathologies may drive prefrontal-subcortical microvascular injury, jointly impairing cognitive-motor integration—a hallmark of MCR pathophysiology (Han and Wang, 2025). Future studies should prioritize longitudinal designs with multimodal phenotyping (vascular dysfunction, chronic inflammation, metabolic dysregulation, and neuroendocrine activation) to disentangle these complex pathways. MCR-S serves as a pragmatic tool for longitudinal investigations. Given its strong validity, the Chinese MCR-S could be integrated into CAD patient screenings in hospitals and clinics, allowing early cognitive assessments and timely interventions. For patients with MCR-S scores exceeding 4.6, it is recommended to continuously monitor changes in cognitive function timely medication adjustments, actively manage cardiovascular risk factors, and implement necessary cognitive training.

### 4.4 Innovations and limitations

The innovations of our study are that, for the first time, we validated the MCR-S in a Chinese population and in a population with CAD, secondly, although the MCR was studied in the elderly (WHO standard for the elderly in developing countries is ≥ 60 years old), due to the increasing youthfulness of patients with CAD and midlife cardiac structure and its change are associated with lower cognition (Rouch et al., 2022; van Gennip et al., 2024), we expanded the age restriction and adjusted the age of the population to those aged 45 years and above. Separate reliability and validity tests were conducted on samples aged 65 years and older (n = 155), and similar conclusions were reached, demonstrating that the scale can be used with middle-aged and older populations. Thirdly, we combined the Rasch and the traditional analyses to test the reliability and validity of the MCR-S, which is a more comprehensive validation method. Our study demonstrated that the MCR-S is effective in validating the MCR-O, which is consistent with previous studies, secondly, we defined the diagnostic cutoff value for the MCR-S in the patients with CAD, and since the original study adjusted for chronic diseases such as diabetes, hypertension, et al. as a covariate (Ayers et al., 2022), the cutoff value from our study may be applicable to the diagnosis of patients with other chronic diseases. However, we do not recommend applying the scale only to patients with a baseline diagnosis of MCR, which is consistent with the original authors’ recommendations (Ayers et al., 2022). Secondly, we recommend expanding the MCR-S items as well as giving clearer definitions of the items or responses to further improve the reliability of the scale.

There are some limitations to the studies we report. One major limitation is that the study is limited to CAD patients, which raises uncertainty about whether the findings can be generalized to the general elderly population or to individuals with cardiovascular risks but no CAD. Additionally, since MCR-S relies on self-reported symptoms, mood and literacy levels may affect responses, necessitating further validation. Furthermore, the study does not examine whether MCR-S scores predict cognitive decline over time. Because we downwardly adjusted the age of the population, the incidence of MCR in the CAD group was lower than in the community population in China (Han and Wang, 2025; Zhang et al., 2020) readers need to be cautious about the conclusion of this rate of occurrence. To enhance generalizability, future research should validate the scale in diverse populations, establish standardized item definitions, longitudinal assessments to determine its predictive validity for cognitive decline/dementia in CAD patients and mechanistic exploration of distinct neurocardiac pathways.

## 5 Conclusion

The Chinese version of MCR-S meets the requirements of the Rasch model and has good validity in CAD patients. The validated MCR-S cutoff can support long-term monitoring and early intervention for CAD patients at risk of MCR-O.

## Data Availability

The original contributions presented in the study are included in the article/Supplementary Material, further inquiries can be directed to the corresponding author.
