# Supplementary material for: Chinese validation of “subjective motoric cognitive risk syndrome” screening tool in patients with coronary artery disease using Rasch analysis
Source: Front Aging. 2025 May 15;6:1505847. doi: 10.3389/fragi.2025.1505847 (PMC12119596; doi:10.3389/fragi.2025.1505847)

Supplementary Material

**Supplemental Material Table 1.** The translation process flowchart


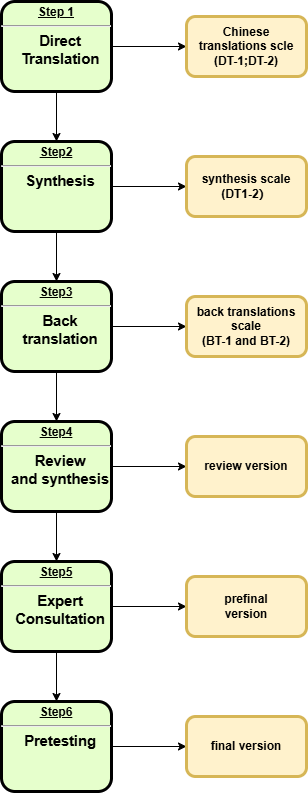


**Supplemental Material Table 2.** English version and Chinese version of the MCR-S.


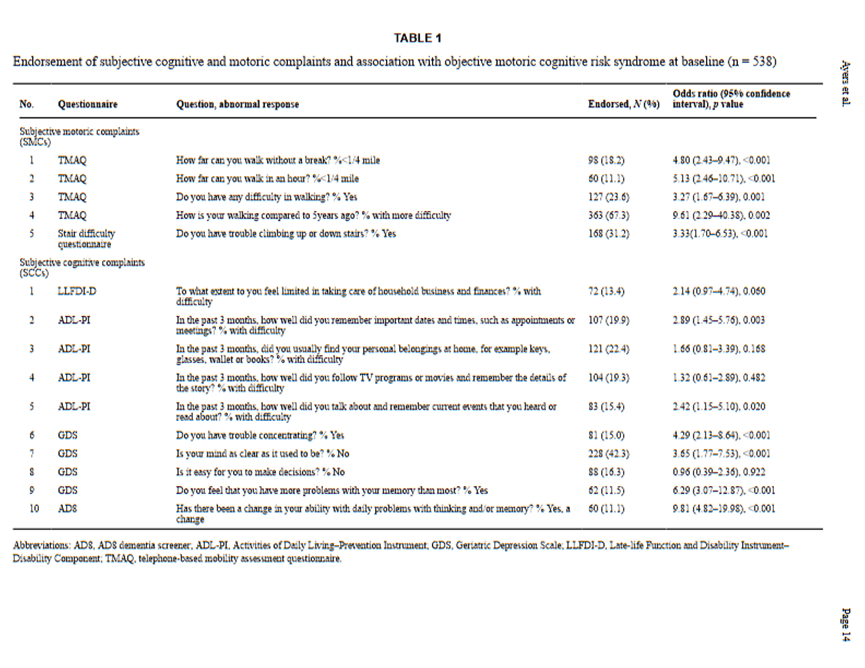

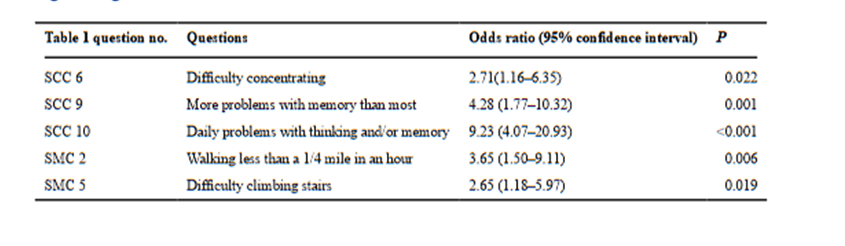


| **Original English version of the MCR-S** |
| --- |
| Do you have trouble concentrating?（SCC6） |
| Do you feel that you have more problems with your memory than most?（SCC9） |
| Has there been a change in your ability with daily problem with thinking and/or memory?（SCC10） |
| How far can you walk in an hour? ＜1/4mile(SMC2) |
| Do you have trouble climbing up or down stairs?（SMC5） |

| **中文版MCR-S问卷** | | |
| --- | --- | --- |
|  | 条目 | 回答及评分 |
| 认知主诉 | 1.您是否难以集中注意力？ | 否（0分） □  是（1分） □ |
|  | 2.您是否觉得您的记忆力比大多数人差？ | 否（0分） □  是（1.5分） □ |
|  | 3.您日常思考和（或）记忆能力是否出现了问题？ | 否（0分） □  是（2.2分） □ |
| 运动主诉 | 4.您一个小时能走多远？是否＜1000米 | 否（0分） □  是（1.3分） □ |
|  | 5.您上下楼梯是是否有困难？ | 否（0分） □  是（1分） □ |
| 总分 | |  |

**Supplemental Material Table 1.** Standardized residual variance in Eigenvalue units

|  | Eigenvalue | Observed | | Expected |
| --- | --- | --- | --- | --- |
| Total raw variance in observations | 9.7 | 100.0% |  | 100.0% |
| Raw variance explained by measures | 4.7 | 48.3% |  | 47.8% |
| Raw variance explained by persons | 2.0 | 21.1% |  | 20.9% |
| Raw Variance explained by items | 2.6 | 27.2% |  | 26.9% |
| Raw unexplained variance(total) | 5.0 | 51.7% | 100.0% | 52.2% |
| Unexplained variance in l^st^ contrast | 1.5 | 15.7% | 20.3% |  |
| Unexplained variance in 2^nd^ contrast | 1.3 | 13.2% | 25.5% |  |
| Unexplained variance in 3^rd^ contrast | 1.1 | 11.5% | 22.3% |  |
| Unexplained variance in 4^th^ contrast | 1.0 | 9.9% | 9.1% |  |
| Unexplained variance in 5^th^ contrast | 0.1 | 1.4% | 2.7% |  |

**Supplemental Material Table 2.** Inter-item residual correlations

| ITEM | ITEM | CORRELATION |
| --- | --- | --- |
| 1 | 2 | -0.2940 |
| 1 | 3 | -0.1743 |
| 1 | 4 | -0.0930 |
| 1 | 5 | -0.0108 |
| 2 | 3 | -0.3222 |
| 2 | 4 | -0.1878 |
| 2 | 5 | -0.2242 |
| 3 | 4 | -0.1708 |
| 3 | 5 | -0.3147 |
| 4 | 5 | 0.0114 |

**Supplemental Material Table 3.** Rasch model separation index and reliability

|  | Measure | Error | INFIT | | OUTFIT | | SI | Reliability |
| --- | --- | --- | --- | --- | --- | --- | --- | --- |
|  |  |  | MNSQ | ZSTD | MNSQ | ZSTD |  |  |
| Person | -1.11 | 1.15 | 1.00 | -0.2 | 1.29 | 0.2 | 1.03 | 0.52 |
| Item | 0.00 | 0.13 | 0.88 | -1.6 | 1.87 | 2.7 | 15.61 | 1.00 |

**Supplemental Material Figure 1.** Study Participants


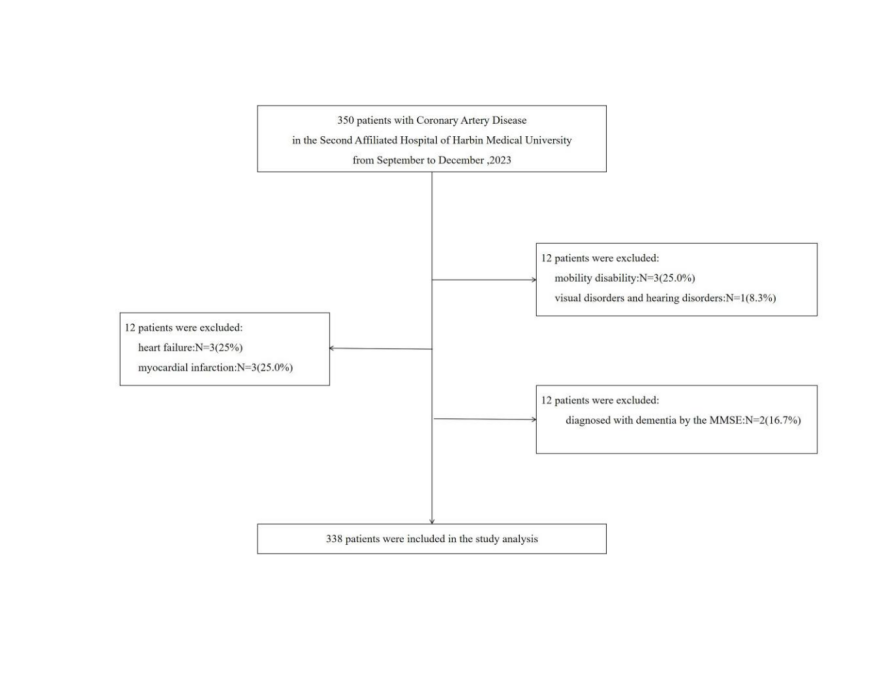


**Supplemental Material Figure 2.** Category probability curves of 5 items.


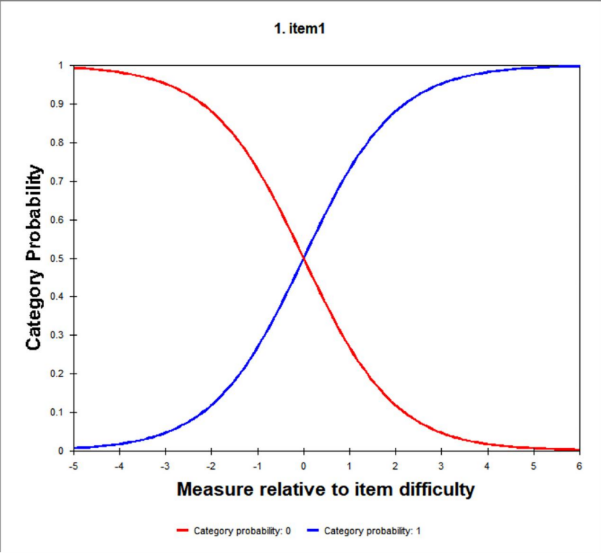

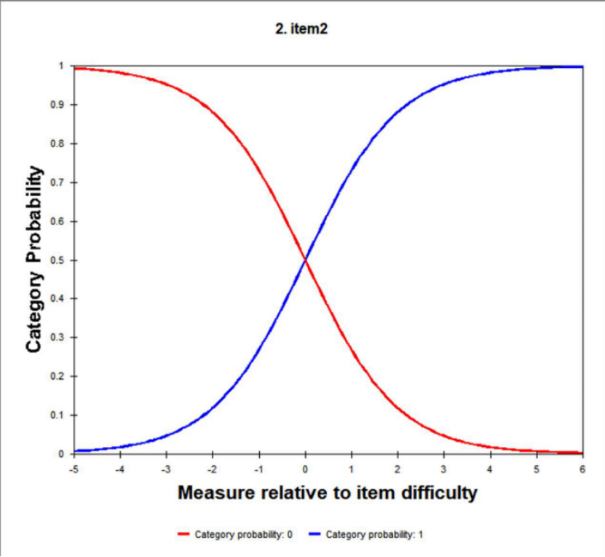

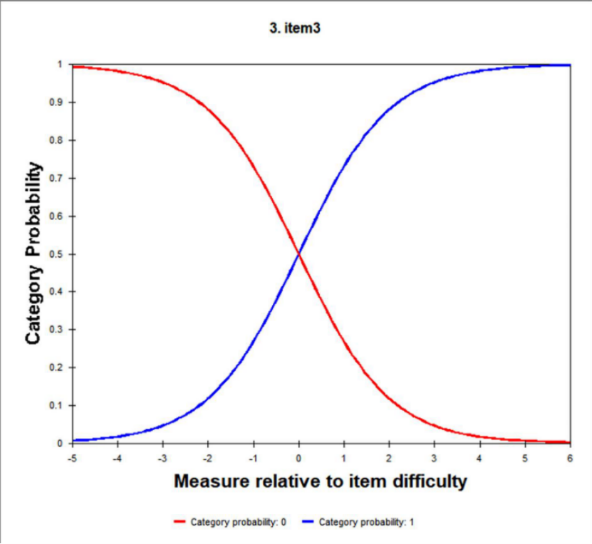

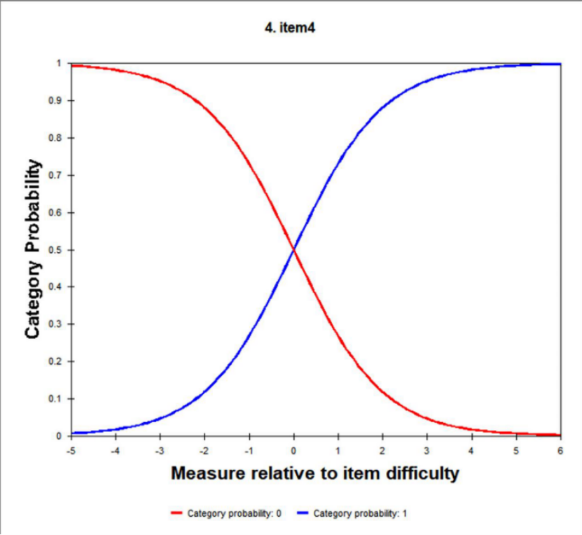

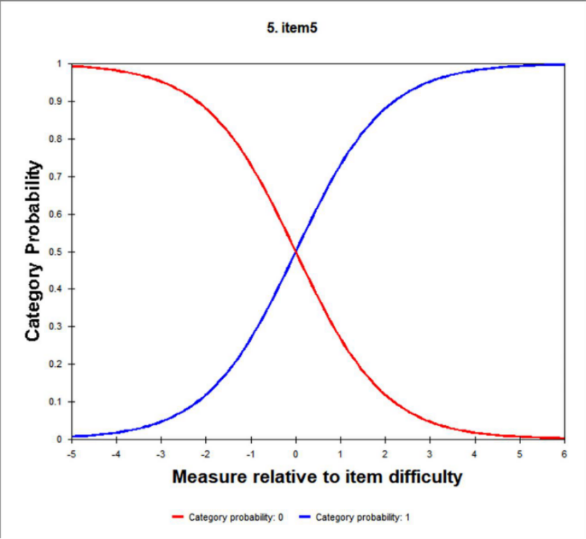


**Supplemental Material Figure 3.** Item Characteristic Curve of 5 items


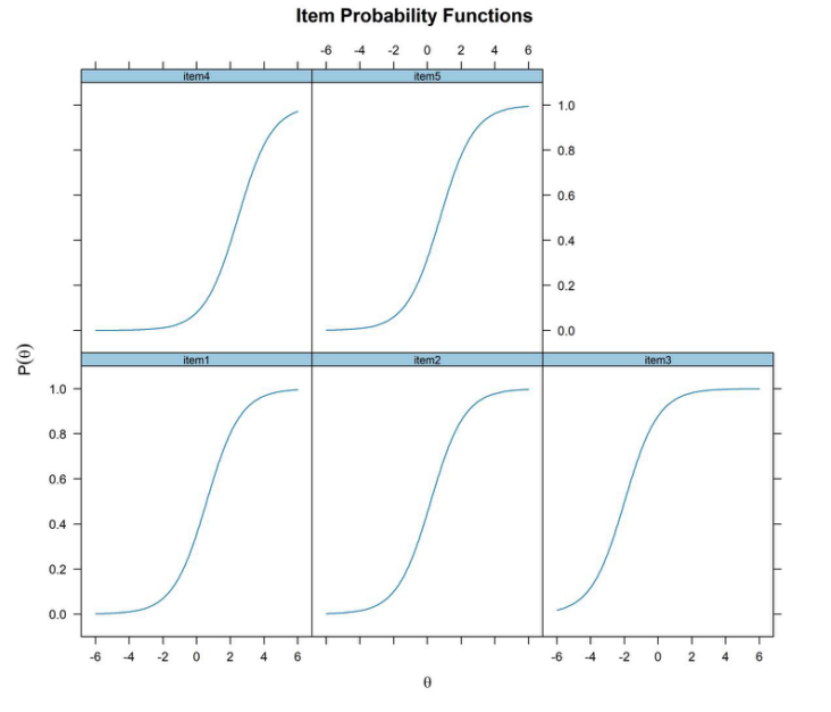

Supplement: Supplementary file 1 [file DataSheet1.docx]
